# Oral Sucrosomial® iron versus intravenous iron for recovering iron deficiency anaemia in ND-CKD patients: a cost- minimization analysis

**DOI:** 10.1186/s12882-020-01716-w

**Published:** 2020-02-22

**Authors:** Eleonora Riccio, Massimo Sabbatini, Ivana Capuano, Angela Maria Pellegrino, Luigi Annicchiarico Petruzzelli, Antonio Pisani

**Affiliations:** 1 Department of Nephrology, University of Campania “Luigi Vanvitelli”, via S. Pansini 5, 80131 Naples, Italy; 2 Chair of Nephrology, Department of Public Health, University Federico II of Naples, Naples, Italy

**Keywords:** Cost-minimization analysis, Oral sucrosomial iron, Intravenous iron, Anaemia, Iron deficiency

## Abstract

**Background:**

Oral iron is recommended as first line treatment of anemia in non-dialysis chronic kidney disease (ND-CKD) patients. Sucrosomial® iron, a new generation oral iron with high absorption and bioavailability and a low incidence of side effects, has shown to be not inferior to intravenous (IV) iron in the replacement of iron deficiency anemia in patients with ND-CKD. Besides the clinical benefit, it is also important to determine the comparative total costs of oral versus IV iron administrations. The aim of this study was to perform a cost-minimization analysis of oral Sucrosomial iron, compared with IV iron gluconate from an Italian societal perspective.

**Methods:**

Cost analysis was performed on the 99 patients with ND-CKD and iron-deficiency anemia of the randomized trial by Pisani et al. Human and material resources utilization was recorded during each iron administration. According to study perspective, direct and indirect costs were considered. Costs for each resource unit were taken from official Italian sources. Probabilistic sensitivity analyses were carried out to test the robustness of the results.

**Results:**

The base case analysis showed an average cost/cycle per patient of € 111 for oral iron and € 1302 for IV iron. Thus, the potential saving was equal to € 1191 per patient/cycle. The sensitivity analysis showed that the most sensitive driver is the time loss by patient and caregivers for the therapy and related-care, followed by the minutes of nursing care and the number of kilometres travelled to reach the referral centre.

**Discussion:**

This study showed that oral Sucrosomial® iron could offer specific advantages in terms of potential savings, and allowed identifying some implications for future research. Such advantages still persist with the new single dose IV iron formulation available in the market, although to a lesser extent.

## Background

Anemia and iron-deficiency are common complications of chronic kidney disease (CKD). Anemia is associated with increased mortality and cardiovascular (CV) events and decreased physical health and quality of life [1, 2]. Although the main cause of anemia in CKD is the relative deficit of renal production of erythropoietin (EPO), iron deficiency plays a crucial role in its genesis [3]. In fact, the latest anemia guidelines from the Kidney Disease Improving Outcomes (KDIGO) initiative recommend to start treatment with Erythropoiesis-Stimulating Agents (ESA) after correction of iron deficiency, and that iron treatment may be performed also in patients with a normal iron balance to increase Hemoglobin (Hb) levels [4]. The optimum route of administration of iron in CKD patients is still controversial. While the weight of up to date clinical evidence generally indicates that the use of intravenous (IV) iron in patients with CKD is more effective than oral treatment, in non-dialysis chronic kidney disease (ND-CKD) there is no widely accepted consensus on whether IV or oral iron should be used as first-line therapy [5]; indeed, a recent report by European Medicines Agency (EMA) (September 2013) clearly points out that IV iron should be prescribed only when oral iron cannot be given or does not work (EMA/579491/2013). However, despite the potential benefits of oral iron, that include easy administration, its use is limited by poor gastrointestinal absorption and high rate of adverse events [6, 7].

Sucrosomial iron (Sideral® Forte), a preparation of ferric pyrophosphate conveyed within a phospholipid and sucrose esters of fatty acid membrane, is a new generation of oral iron, which shows a high intestinal absorption and high bioavailability with a low incidence of side effects, due to lack of any direct contact with intestinal mucosa [8–10]. In comparison with the other standard oral iron preparations, sucrosomial iron seems to be a promising new strategy of iron replacement in several kinds of patients [11–13], and in particular in ND-CKD patients [8].

A recent, randomized clinical trial performed in patients with ND-CKD and iron-deficiency anemia showed that Sucrosomial iron was non-inferior to a typical dosing strategy of IV iron gluconate with regard to the primary efficacy end point, the mean change in Hb values from baseline to the end of treatment [8]. Although the short-term therapy with IV iron produced a greater replenishment of iron stores and a more rapid Hb increase compared with sucrosomial iron, the final increase in Hb values was similar with either treatment; moreover, after iron withdrawal, Hb concentrations remained stable in Group IV, while recovered to baseline in oral group. The incidence of adverse event was significantly lower in the oral group, and the adherence was similar in the two groups [8]. Besides the clinical benefit, it is also important to determine the comparative total costs of oral versus IV administrations. Based on a within-trial analysis of the results of study, aim of this paper was to compare the cost implications of Sucrosomial® iron in comparison with IV iron from an Italian societal perspective.

## Methods

### Study design

The study was designed as a cost-minimization analysis of oral Sucrosomial® iron, compared with IV iron, based on primary data collected during the Phase III study by Pisani et al. [8]. The methods and efficacy results of the clinical study have been described in detail previously [8]. Briefly, this randomized study aimed to determine whether oral Sucrosomial® iron, compared with IV iron, improves anemia in ND-CKD patients. 99 patients with ND-CKD and iron-deficiency anemia were randomized (2,1) to receive oral Sucrosomial® iron (one oral capsule/day containing 30 mg of sucrosomial iron and 70 mg of ascorbic acid - Sideral® Forte, Pharmanutra Spa; 66 patients) or a total dose of 1000 mg of IV iron gluconate (125 mg diluted in 250 mL normal saline, infused weekly, 33 patients). The patients were followed-up for a 3-months treatment period and the results showed that oral Sucrosomial® iron was a safe and efficacious alternative to IV iron gluconate to correct anemia in ND-CKD patients.

Economic analysis was conducted on the 99 patients that completed the study trial. A cost-minimization analysis was performed to estimates the potential savings deriving from oral iron therapy versus IV iron treatment. Total costs were calculated as sum of direct and indirect costs occurred during the clinical study. Average cost per patient/cycle was calculated by dividing total costs per number of patients in each group. Potential saving per patient/cycle was calculated as the difference between the average cost per patient/cycle in the IV iron group and the average cost per patient/cycle in the oral iron group.

### Base case analysis

To estimate the costs of iron administration, an Activity Based Costing approach [14] was followed. Resource utilization was obtained from the clinical study and was reported in Table 1. According to a wide societal perspective adopted, resources from patients, their families (informal care) and third-party payers were considered to calculate direct and indirect costs related to iron therapy.

**Table 1:** Resources for patient/administration

Resources	IV iron	Oral iron
Nephrologist (n)	1	–
Nurse (n)	1	–
NaCl 0,9% (ml)	250	–
Disposable materials (needle, siringe, intravenous line, patch, cotton)	1 per product (0.25)	–
Iron administration	10.03 (0.39)	89.2 (1.63)
Min nursing time (dev. std)	45 (11.2)	–
Min Clinician time (dev.std)	20 (5)	–
Min time losses for patients and caregivers (dev.std)	178 (44.5)	–
Km of average distance from patients’ home (dev.std)	21.8 (19.6)	–

Since oral iron is self-administered by the patient and is, therefore, not associated with any administration costs, besides those of the medication, resources measurement focused on IV iron administration. Two physicians assisted each iron infusion and measured times, material and human resources uptake directly associated with IV iron treatment. The crucial phases of the iron administration were identified by analyzing data collected and by consulting two key opinion leader nephrologists. A questionnaire was administered to collect data on possible adverse events [8] and to quantify indirect costs (Supplementary material). We asked patients to report if they were workers and if they were accompanied to the hospital. We also asked about the distance travelled from patients’ home (expressed in kilometers) and about the time taken to reach the hospital. Only costs occurring during the 3 months treatment period were included in the analysis. Laboratory tests costs were excluded from the present analysis since all patients of both groups underwent the same diagnostic tests. Trial-related follow-up visits in both treatment arms and related resource use have not been considered for the same reason. Costs for each resource unit were taken from official Italian sources (Table 2). All costs were calculated in Euro.

**Table 2:** Unit costs and costs sources

	Unit cost	Sources
Nurse hourly cost	€ 31	Federico II University Hospital accounting office
Nephrologist hourly cost	€ 52	
Materials	€ 0.73	Referral prices 2016 [15]
IV iron (1 pack. 312,5 mg)	€ 4.36	Ex-Factory Price, AIFA 2014 [16]
Sucrosomial Iron (1 pack. 600 mg)	€ 24.90	Public purchase price
Transportation cost/km	€ 0.30	ACI GU 2018 [17]
Productivity loss/hour	€ 28	Eurostat 2016 [18]
Hospitalization cost	€ 260	DRG 316 [21]

IV iron costs were based on the actual usage in the clinical trial and calculated on the basis of ex-factory prices as reported by Italian Agency of Drug (AIFA) [16]. Material costs were derived from referral national prices [15]. Medical and nursing care (expressed in minutes) were observed and measured for each patient. Personnel wages were derived from the hospital accounting office. The resulting hourly costs were multiplied by the mean administration time. Productivity losses were estimated using the human capital method [18, 19]. The loss of production costs represents the cost of illness in social terms. Productivity losses refers to indirect costs due to absence of employer from work. Workers often lost wages and employers must deal with decreases in production output when employees are not at work because their illness. The human capital method monetizes the value of productivity losses, by considering the patient’s hours of productivity that are lost and calculates productivity costs as the product of those total lost hours with the hourly wage, that we derived from Eurostat website [18, 19]. Transportation costs were estimated based on cost/km derived from official Italian tables [17], by considering only one-way kilometers travelled to reach the hospital. Furthermore, we considered an overhead, including general hospital services, of 10% of total costs. Unit costs and their sources are shown in Table 2.

### Scenario analysis

A deterministic scenario analysis was conducted to determine the potential saving related with oral iron use in a different setting. A hypothetical scenario was built to reflect an ideal clinical practice setting assuming 1) a perfect patient adherence to the treatment and 2) the same treatment efficacy observed in the trial. Mean resource use was derived by interviewing the trial investigators. As clarified by EMA and by AIFA, IV iron should be administered in a hospital setting where personnel, specifically trained, can treat eventual allergic reactions [20]. Therefore, in this case, healthcare resource utilization refers to outpatient visits (ten/cycle for IV iron and one/cycle for oral iron patients) and to day-hospital or day service-related cost drivers. Unit costs for specialist visits, equal to € 20,66 each, were obtained from the National Pricing System updated by the Ministerial Decree in 2012 [21]. Diagnosis-related group (DRG) tariff for Day-hospital [21] were used as proxy of day service tariffs, since they are not uniquely defined at national level. The nephrology day-hospital tariff results equal to € 260 from DRG 316.

### Sensitivity analysis

Sensitivity analysis aims to test the uncertainty of the study results to improve research methods and to obtain robust results to inform decision making process [22]. A one-way sensitivity analysis allows to examine the impact on results of each variation of the drivers considered. To test the consistency of the findings of this study, a one-way sensitivity analysis was performed by assuming an inverse normal (Inv-norm) random variable and by using 10th percentile and 90th percentile to identify minimum and maximum values of inputs’ variation. This approach was used to vary the time variables (medical and nursing time, time loss), distance kilometres, material use and number of IV infusions or oral administrations. The most sensitive parameters were reported in the tornado diagram. Moreover, a multivariate probabilistic sensitivity analysis was performed by varying all base case cost-inputs simultaneously, according to a gamma random variable. Results were reported in a probabilistic cost-saving curve.

## Results

### Base case analysis

As described in the study by Pisani et al., patients of two groups of iron therapy were comparable for age, sex, body weight, estimated glomerular filtration rate (eGFR) and use of ESA (Supplementary Material). Patients randomized in the oral Sucrosomial® iron arm (66 patients, age 53.1 ± 15.0 years; 73% females) received on average 89 administrations each one (1 cycle), including one capsule/day. Patients randomized to the IV iron group (33 patients, age 47.6 ± 16.0 years; 70% females) received on average 10,03 injections of IV iron over the course of the study (one cycle therapy). Each infusion of the 33 patients involved one nephrologist and one nurse in an inpatient setting: IV iron was handled by the nurse and required about 45′ of nursing time for each administration; furthermore, the infusion was also supervised by a Nephrologist, on the average for 20 min.

The material needed for each IV iron infusion consisted of: one injection needle, one intravenous line, one patch, a cotton and 250 mL 0,9% NaCl. In contrast, oral iron was self-administered by the patient and was, therefore, not associated with any administration cost, except for treatment costs.

The average distance travelled from patient’s home to receive the IV iron administration was estimated at about 22 km (one way), resulting in an accumulated average travel distance of 219 km per patient during the study period. The whole IV therapy for one patient was estimated to take 3,3 h per injection, including travel time. Patients were accompanied in 9 out of 33 cases and companions were usually younger and healthy people; therefore, an estimated cost for loss of work time by caregivers was also included in the economic comparison. Resource use for each iron administration and standard deviation are reported in Table 1. The cost components used in the present study are described together with their unit costs in Table 2.

The cumulative cost related to oral iron supplementation, which was given only by the cost of the product itself, was of € 7329.32, and the average cost was equal to € 111.05 per patient/cycle. Oral iron therapy is not associated with administration or indirect costs, so the resources consumption refers to the cost per mg multiplied by the number of average mg per patient/cycle registered during the study trial. Conversely, the resources consumption related to IV iron injections was equal to € 577, for an average cost per patient of € 17,50 per cycle. Material resources used during every cycle generate a cost of € 7.3 per patient. Hourly costs of nurses and clinicians resulted in € 31 and € 53, respectively, for an average cost per cycle of nursing care of € 234 and €177 for medical care. Travel costs were estimated for € 65.75 per patient/cycle.

Regarding the indirect costs, 21 out of 33 patients were employed, resulting in € 530 of productivity losses per patient/cycle. Caregivers’ productivity costs were estimated equal to € 227 (on average).

Thus, considering the average cost per patient/cycle of € 1302.30 for IV iron and € 111.05 for Sucrosomial iron, the potential saving was equal to € 1191.25 per patient/cycle (Table 3).

**Table 3:** Base case analysis - Average costs per patient/cycle

	IV iron	Sucrosomial Iron
*Direct healthcare costs*		
Drugs	€ 17.49	€ 111.05
Materials	€ 7.38	
Medical care	€ 176.83	–
Nursing care	€ 233.85	–
Overhead	€ 43.56	–
Total direct healthcare costs	**€ 479.11**	**€ 111.05**
*Not-medical direct costs*		
Transportation	€ 65.75	–
Total not-healthcare costs	**€ 65.75**	–
*Indirect costs*		
Productivity losses –patients	€ 530.21	–
Productivity losses – caregivers	€ 227.23	–
Total indirect costs	**€ 757.44**	
Average cost per patient/cycle	**€ 1, 302.30**	**€ 111.05**
Potential saving per patient/cycle	**€ 1191.25**	

This study did not consider different iron formulations like iron sucrose, dextrane, or carboxymaltose, which can be administered at high doses in single visits, because of their low availability in Italy at time of Pisani’s study. Nevertheless, a hypothetical analysis based on the cost of a 1 g administration of iron carboixymaltose (currently available in Italy) in 1 or 2 infusions still remains more expensive than one cycle of oral iron administration of Sucrosomial iron. In fact, on the basis of the public cost of the drug and considering the same accessory costs of the previous study (materials, medical and nursing assistance) the total cycle cost should grossly range between € 430 and € 590, with 1 or 2 administrations, with an approximate saving of 319 and 479 euros, respectively.

### Scenario analysis

In the scenario analysis, the potential saving was calculated based on mean potential resources use in a hypothetical clinical practice scenario. Direct healthcare costs refer to resources uptake from a third-party payer. In this case, the oral iron costs included also the outpatient visit costs in addition to drugs costs. For the IV iron group, outpatient visits costs and day hospital costs were considered as direct costs. In an ideal clinical practice scenario, the potential saving was € 1117.39 per patient/cycle (Table 4).

**Table 4:** Scenario analysis- Average costs per patient/cycle

	IV iron	Sucrosomial Iron
*Direct healthcare costs*		
Drugs		€ 111.05
Outpatient visits	€ 207.23	€ 61.98
DH/DS	€ 260	–
Total direct healthcare costs	**€ 467.23**	**€ 173.03**
*Not-medical direct costs*		
Transportation	€ 65.75	–
Total not-healthcare costs	**€ 65.75**	–
*Indirect costs*		
Productivity losses –patients	€ 530.21	–
Productivity losses – caregivers	€ 227.23	–
Total indirect costs	**€ 757.44**	
Average cost per patient/cycle	**€ 1, 290.42**	**€ 173.03**
Potential saving per patient/cycle	**€ 1117.39**	

### Sensitivity analysis

**Fig. 1:**
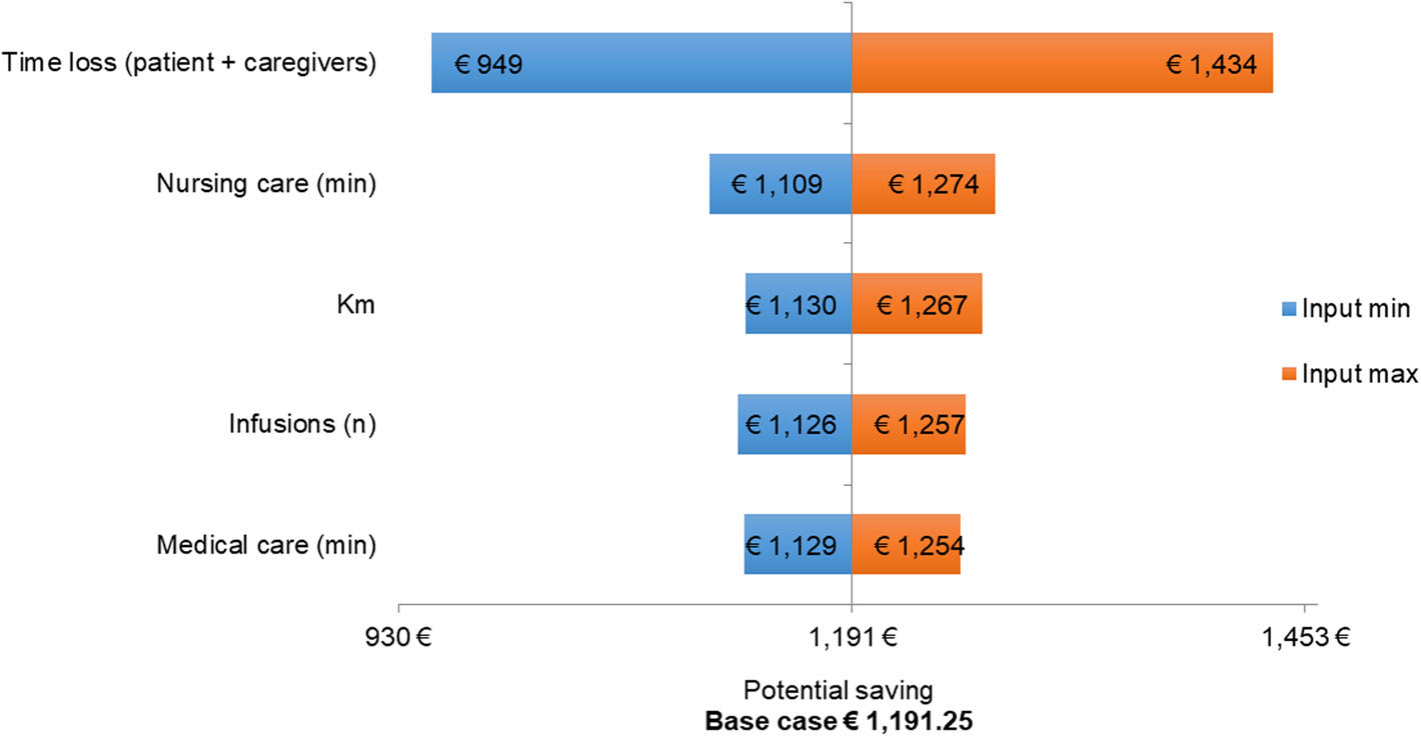
The tornado chart summarizes the results of sensitivity analysis when driver costs were varied

**Fig. 2:**
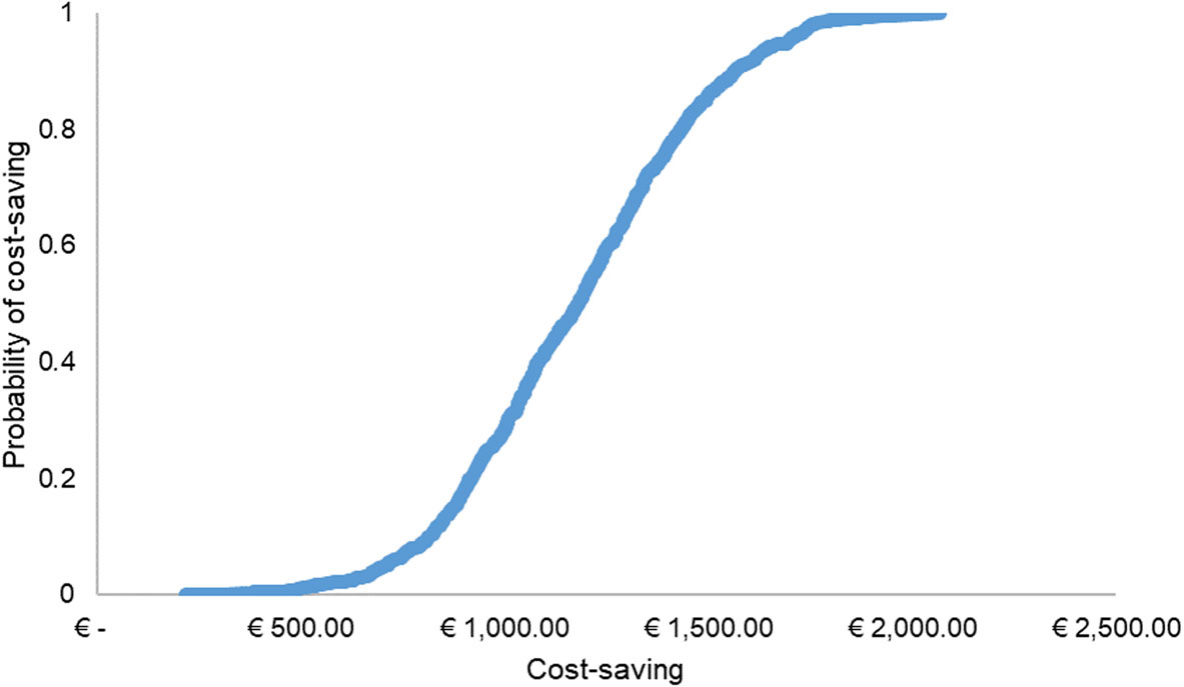
Potential cost-saving projected according to a probabilistic distribution

The sensitivity analysis showed that the most sensitive driver is the time loss by patients and caregivers for the therapy and related-care, followed by minutes of nursing care and number of kilometers travelled to reach the referral center. Finally, the number of IV infusions and medical care are the least sensitive drivers. By varying nursing care minutes (our cost input), the potential saving changes from €1109 to € 1274, when the input is minimum and maximum respectively. The variation of medical care time does not impact significantly (from €1129 to €1254 vs base case of €1191). Variations of materials and number of oral administrations seem to not have an impact on the study results. The tornado chart depicted in Fig. 1 summarizes the results of sensitivity analysis when driver costs were varied. The Fig. 2 represents the potential cost-saving projected according to a probabilistic distribution. In particular, the curve shows a range of saving costs included between € 400 and € 2000, related to minimum and maximum potential saving, respectively.

## Discussion

This study was aimed to estimate the potential saving associated with oral Sucrosomial® iron versus IV ferric gluconate iron administration for treatment of iron deficiency anemia in ND-CKD patients. Since the social perspective adopted, the present study provided a comprehensive overview of healthcare and social care resources related to iron administration to these patients.

The economic study focused on the resources used in the trial by Pisani et al., that demonstrated the same efficacy of oral iron and IV iron, includes direct and indirect costs, with a further analysis to quantify such costs in a real clinical practice scenario; this study represents the first cost analysis carried out on oral Sucrosomial® iron in the treatment of sideropenic anemia in patients with ND-CKD. Administration of Sucrosomial® iron could lead to a potential saving up to € 1191 per patient/cycle in comparison with IV iron treatment, from a wide societal Italian perspective.

Even if the out-of-pocket expenses (direct for patients) in the oral iron group resulted higher than those in IV arm (€ 111 per cycle vs. € 65.75), different cost categories should be considered. First, the expenses for transportation should be double of those reported, since we considered only one-way travel costs; second, other “intangible” costs played a relevant role in this analysis that, although difficult to quantify, may consistently amplify the difference between the oral (and easier) drug administration and the IV (and riskier) drug infusion. Moreover, IV treatment certainly impacts patients’ quality of life and health service costs: all the infusions, in fact, were provided in a hospital setting, with a defined maximum dose that obviously influenced the frequency of patients’ visits up to 3 times/week. Furthermore, because of the risks related to IV route, iron infusions require medical supervision and nursing monitoring and may be performed only in authorized centers equipped with emergency and intensive care units, with scheduled appointments. This obviously results in longer time losses and higher informal care.

Today, the clinical scenario of iron balance management has deeply changed, thanks to the introduction in the market of new IV iron preparations, like ferric carboxymaltose (FCM) that can be administered in high doses (500–1000 mg) in a single IV administration with acceptable side effects, although in-hospital administration is still requested [23]. This study did not consider in the analysis this “comparator”, scarcely available in Italy when the study by Pisani et al. was performed; as shown, however, even a single administration of 1 g of carbxymaltose iron should hypothetically cost 74% more than the entire oral cycle with Sucrosomial iron, suggesting that the oral route still remains the most convenient option, at least from an economic point of view; on the other hand, considering the consistent reduction in costs and, mostly, in personnel involvement, the recourse to FCM, widely available on the market, may more easily find a specific indication when oral iron administration cannot be pursued, mostly considering the savings that a correct iron balance allows on costs of ESA treatment and on the days of hospitalization or of work- hour loss that anemia may require. So, actually, the problem is to understand how much the society would be willing to pay to afford the costs of the IV iron treatment [14, 24].

The study has some limits; the first is related to the representativeness of the sample enrolled in the clinical study supporting the efficacy of Sucrosomial® iron. The economic analysis is based on data from 99 patients of the trial, so it could threaten the generalizability of findings to an Italian reality. We have explored this kind of uncertainty in the probabilistic sensitivity analyses, that allow to verify how input variations could impact on study results, that is what would happen if some aspect of the data or the analysis were changed. Therefore, the robustness of the study was observed by exploring the variability of each used resource with the one-way sensitivity analysis and by reporting results on tornado diagram. Furthermore, it was conducted a multivariate probabilistic sensitivity analysis that produces a range of potential savings based on a casual probabilistic distribution in order to make study results more representative. Sensitivity analyses showed that study results are quite robust.

Another limitation of our study refers to the deterministic scenario analysis that is based on some assumptions. Firstly, we considered the effectiveness of oral iron equal to the efficacy demonstrated in the clinical trial, even if the drug efficacy may be different depending on study setting, under controlled conditions or in the real world. Second, we carried out a deterministic scenario analysis by assuming an average resource use as reported by clinical expert opinion leader, without exploring its variability. Finally, we assume that the patients of the experimental and controlled study have the same behaviors of patients of the real clinical practice. Obviously, it should be also taken into account that the study was carried out in an Italian setting. Moreover, the study reflects costs, laws, and organization related to Italian Ministry of Health, and do not necessarily apply to different countries.

Our cost minimization analysis is based on the main clinical trial result, that is the demonstrated same efficacy of oral iron and IV iron. Sucrosomial iron has demonstrated a similar effectiveness, with lower costs, in patients usually receiving IV iron (e.g., chronic kidney disease, cancer, bariatric surgery) in several studies published recently [25–28]. In particular, Darbà et al. [26] conducted a budget impact analysis of an oral sucrosomial iron in the treatment of patients similar with those of our study. They conclude that increase in the use of oral sucrosomial iron leads to overall budget savings of €775,464 for the Spanish National Health Service over 4 years. A retrospective study [28] evaluated the efficacy of preoperative Sucrosomial iron in 200 paired-matched patients undergoing prosthetic hip surgery reports that Sucrosomial iron supplementation led to a quicker post-surgical recovery, shorter hospitalisation and decreased surgery-related costs with an estimated cost saving of 1763/patient.

## Conclusions

In conclusion, the results of our analysis suggest that administration of oral Sucrosomial® iron may represent a cost-saving option compared with the most likely alternative existing therapy used for the management of iron deficiency anemia in ND-CKD patients in Italy. Future search should focus on related intangible costs and explore effectiveness and costs of oral Sucrosomial® iron in a real clinical practice, maybe compared with other different therapies used for anemia treatment in ND-CKD patients.

## Supplementary information

**Additional file 1.**

## Data Availability

The datasets used and/or analysed during the current study are available from the corresponding author on reasonable request.

## Abbreviations

AIFA: Italian Agency of Drug
CKD: Chronic kidney disease
CV: Cardiovascular
DRG: Diagnosis-related Group
eGFR: Estimated glomerular filtration rate
EMA: European Medicines Agency
EPO: Erythropoietin
ESA: Erythropoiesis stimulating agents
FCM: Ferric carboxymaltose
Kb: Hemoglobin
KDIGO: Kidney disease improving outcomes
ND-CKD: Non dialysis chronic kidney disease
