# Supplementary material for: Oral Sucrosomial® iron versus intravenous iron for recovering iron deficiency anaemia in ND-CKD patients: a cost- minimization analysis
Source: BMC Nephrol. 2020 Feb 22;21:57. doi: 10.1186/s12882-020-01716-w (PMC7035732; doi:10.1186/s12882-020-01716-w)
Supplement: Supplementary file 1 — Additional file 1. [file 12882_2020_1716_MOESM1_ESM.docx]

**1. QUESTIONNAIRE FOR INDIRECT COSTS:**

1. Are you a worker?
   - Yes
   - No
2. Are you retired?
   - Yes
   - No
3. Have you been accompanied today for the iron infusion?
   - Yes
   - No
4. Does your companion work?
   - Yes
   - No
5. How far is your home from the hospital? (Kilometer)
   - ……………………………………….
6. How did you reach the hospital?
   - By car
   - By bus
   - By train
   - Other…………………………….
7. How long did it take you to reach the hospital?
   - ………………………………………….

**2. QUESTIONNAIRE FOR ADVERSE EVENTS:**

During Iron therapy, did you experience one of the following symptoms?

• constipation (<1 bowel movement per 2 days)

• diarrhea (>3 bowel movements per day)

• bloating

• nausea, cramps, sense of indigestion

• muscle cramps

• episodes of low blood pressure

• skin rash

0 = none; 1 = somewhat/occasionally; 2 = a lot/often.

**3. Main characteristics of the two iron treatment group of study by Pisani et al.**

|  | **Group OS (n=66)** | **Group IV (n=33)** |
| --- | --- | --- |
| Age (years) | 53.1±15.0 | 47.6±16.0 |
| Sex (% female) | 73 | 70 |
| Body weight (kg) | 70.5±13.5 | 70.2±16.2 |
| Systolic BP (mmHg) | 132±15 | 131±18 |
| Dialstolic BP (mHg) | 77±7 | 79±7 |
| eGFR (mL/min/1.73m^2^) | 25.9±11.4 | 31.8±12.9 |
| **CKD stage (%):** |  |  |
| Stage 3 (30-60 mL/min/1.73 m^2^) | 43 | 48 |
| Stage 4 (15-30 mL/min/1.73 m^2^) | 49 | 43 |
| Stage 5 (≤15 mL/min/1.73 m^2^) | 9 | 9 |
| **Renal diseases (%):** |  |  |
| GN | 28 | 30 |
| DM | 29 | 26 |
| ADPKD | 16 | 22 |
| Urological causes | 5 | 3 |
| Other/unknown | 22 | 19 |
| **Drug treatments (%):** |  |  |
| ACEi | 45 | 40 |
| ARBs | 20 | 18 |
| ESA | 5 | 4 |

Data are expressed as means ± standard deviation or as percentage and frequency.

Abbreviations: BP, blood pressure; eGFR, estimated glomerular filtration rate; CKD, chronic kidney disease; GN, glomeruolonephritis; DM: diabetes mellitus, ADPKD, autosomal dominant polycystic kidney disease; ACE-I, inhibitors of angiotensin converting enzyme; ARB, angiotensin receptor blockers; ESA, erythropoiesis stimulating agents.
